# Clinical characteristics and outcome of the first 200 patients hospitalized with coronavirus disease-2019 at a treatment center in Abuja, Nigeria: a retrospective study

**DOI:** 10.11604/pamj.2022.41.118.26594

**Published:** 2022-02-10

**Authors:** Vivian Gga Kwaghe, Zaiyad Garba Habib, Alexander Agada Akor, Yunusa Thairu, Anthony Bawa, Francis Olayemi Adebayo, Ayi Vandi Kwaghe, Galadima Usman, Godwin Idoko, Akintola Oluseugun, Bissallah Ahmed Ekele

**Affiliations:** 1 Department of Internal Medicine, University of Abuja Teaching Hospital, Gwagwalada, Abuja, Nigeria,; 2 Department of Microbiology, University of Abuja Teaching Hospital, Gwagwalada, Abuja, Nigeria,; 3 Department of Pediatrics, University of Abuja Teaching Hospital, Gwagwalada, Abuja, Nigeria,; 4 Department of Obstetrics and Gynaecology, University of Abuja Teaching Hospital, Gwagwalada, Abuja, Nigeria,; 5 Nigeria Field Epidemiology and Laboratory Training Programme, Abuja,; 6 Department of Veterinary and Pest Control Services, Federal Ministry of Agriculture and Rural Development, Abuja, Nigeria,; 7 Department of Anaesthesia, University of Abuja Teaching Hospital, Gwagwalada, Abuja, Nigeria

**Keywords:** COVID-19 patients, clinical features, treatment outcome, comorbidities, Nigeria

## Abstract

**Introduction:**

globally, the ravaging effect of the coronavirus disease-2019 (COVID-19), pandemic is evident on public health and the global economy. We aimed at describing the clinical characteristic and management outcome of COVID-19 patients in Abuja, Nigeria.

**Methods:**

we conducted a retrospective study by reviewing the hospital charts of the first 200 COVID-19 patients admitted at the isolation center, University of Abuja Teaching Hospital (UATH), Gwagwalada. Extracted data includes; demographic data, clinical symptoms, underlying comorbidities, and clinical outcomes. The outcome of interest was either discharged or died. Data was analyzed using the Statistical Package for Social Sciences (SPSS) version 20.0.

**Results:**

the median age was 45 years (range 2-84 years). Majority of the patients were males (66.5%). The most affected age group was 50-59 years (21%). Children and adolescents were least affected; less than 10 years constituted 2.5% and 10-19 years constituted 4.5%. The commonest symptoms at presentation were fever (94%) and cough (92%). Ninety-four patients (47%) had underlying comorbidities; the commonest was hypertension (36%). Based on disease severity; 126 (63%) had mild disease, 22 (11%) had moderate disease and 52 (26%) had severe disease. The commonest complication was Acute Respiratory Distress Syndrome (ARDS) seen in 29 (14.5%) patients. Out of the 200 cases managed, 189 (94.5%) were discharged in a stable condition while 11 (5.5%) died. Patients with under lying comorbidities had 9.6% death rate while those without comorbidities had 1.9% death rate.

**Conclusion:**

among Nigerian patients', COVID-19 affects males more than females while children and adolescents were least affected. The study highlighted the clinical features of COVID-19 patients. The overall mortality rate is low among Nigerian patients compared to patients in the USA and Europe. This study shows that advanced age, presence of underlying comorbidities and disease severity is associated with the risk of dying from COVID-19.

## Introduction

The World is being ravaged by the severe acute respiratory syndrome coronavirus-2 (SARS-CoV-2) pandemic; a new strain of coronavirus. The ongoing pandemic started in China in December 2019 but rapidly spread to other parts of the World. It was declared a global pandemic by the World Health Organization (WHO) on March 11 2020 [1]. Globally, about four waves of surge of COVID-19 have occurred. Factors associated with the increase or decline of the disease are; the effectiveness of vaccine over time, human social behaviour, institution of infection prevention policies, evolution of the virus resulting in the creation of new variants and the number of vulnerable people who have not developed some immunity against the virus via natural infection or vaccination [2]. As of January 5^th^, 2022, 295,697,038 cases have been reported worldwide with 256,228,260 recoveries and 5,476,274 deaths [3]. The African continent has 9,970,116 cases, 8,828,727 recoveries and 229,990 deaths by 5^th^ of January, 2022 [3].

Nigeria recorded the first case of COVID-19 on the 27^th^ of February 2020, when an Italian businessman who had returned from Milan, tested positive to the virus [4]. The Country currently has 244,548 recorded cases, 216,814 recoveries and 3,053 deaths as at 5^th^ of January, 2022 [5]. Abuja, the Federal Capital Territory has the 2^nd^ highest number of cases 27,389 with 24,318 recoveries and 239 deaths [5]. Majority of data on clinical characteristics of COVID-19 is from China, Europe and the USA. There is paucity of data on the clinical characteristics of COVID-19 in Africa. Research has shown that there is significant difference in the clinical and demographic characteristics of COVID-19 patients from different parts of the world. Moreover, as a new infectious disease, it is particularly important that clinicians from different parts of the world report its clinical and demographic characteristics as seen in their regions of practice. Several studies on the clinical features and outcome of COVID-19 in other parts of the world and a few in Africa have been conducted [6-11] regarding the clinical presentation have been conducted in Lagos, Nigeria, which authenticates the need for further studies to add to the existing body of knowledge in this area. The research hypotheses were; there are differences in the descriptive epidemiology of clinical signs of COVID-19 patients in Nigeria compared to what was obtained in other countries. The presence of comorbidities increases the chances of death with SARS-CoV-2 infection in Nigeria. The objective of this study is to describe the clinical characteristics and outcome of COVID-19 in Nigeria.

## Methods

**Study site:** the University of Abuja Teaching Hospital (UATH) is located in Gwagwalada, one of the six local councils managed by the Federal Capital Territory (FCT) Authority, Abuja, Nigeria. The hospital is a tertiary facility that serves as a referral hospital to the whole of the Federal Capital Territory (FCT) as well as the neighboring states. The 520 bedded capacity hospital also has a biosafety laboratory level 3 (BSL3). The UATH COVID-19 isolation centre is one among the seven isolation centres located in the Federal Capital Territory (FCT) and was the first hospital in the FCT to manage patients with COVID-19. It is a 42 bedded capacity isolation centre saddled with the responsibility of handling all patients that have moderate to severe symptoms of COVID-19 infections in the FCT. Patients were admitted into the isolation center after they tested positive to COVID-19. Majority of cases were referred from other isolation centres in the FCT to the UATH isolation centre for specialist care.

**Study design and population:** we conducted a retrospective study by reviewing the hospital charts of the first 200 COVID-19 patients admitted at the isolation center, UATH Gwagwalada within the period of March to June 2020. Our inclusion criteria were all patients admitted at UATH Gwagwalada that tested positive to COVID-19 within the study period who have been discharged or died. Our exclusion criteria were patients that tested positive to COVID-19 and that were still on admission at the end of June, 2020. Data from the patients´ health records were retrieved by the clinicians in charge of the patients at the isolation centre with the help of a trained research assistant. All retrieved data were de-identified. Retrieved data were transcribed into a standard format which tallied with the objectives of the study. Extracted data includes; demographic data, clinical symptoms, underlying comorbidities, and clinical outcomes. There were no missing data as all data obtained were from the patients records which had all the demographic characteristics, clinical history, signs/symptoms, diagnostic test conducted and treatment. There was no formal means of sample size calculation since the study is a retrospective study. Study size was determined based on the number of patients that were admitted and discharged or died within the stipulated study period. We extracted data of the first 200 patients from the patients´ medical records. The diagnosis of COVID-19 was done by real-time polymerase chain reaction (RT-PCR) using nasopharyngeal and oropharyngeal swabs [12,13]. We categorized patients into three groups based on severity of symptoms; mild, moderate and severe. Patients who had no shortness of breath and had oxygen saturation greater than 90% were considered to have mild disease. Those with mild shortness of breath and oxygen saturation greater than 90% were categorized as having moderate disease while those with severe shortness of breath with oxygen saturation less than 90% or who had any organ failure were considered to have severe disease. The outcome of interest was either discharged or died. There was no bias associated with this study as all data obtained for the study were from records of patients who have been discharged from UATH isolation centre or those that died from the disease.

**Data analyses:** data was analyzed using the Statistical Package for Social Sciences (SPSS) version 20.0. Continuous variables were expressed as median and range. Categorical variables were analyzed as counts and percentages.

**Ethical approval:** data used for this study was based on secondary data analysis and ethical approval is not required. The UATH hospital management gave the approval for the release and use of the data.

## Results

Majority of the patients were males (66.5%). The median age was 45 years (range 2-84 years). The most affected age group was 50-59 years (21%) (Table 1). Children and adolescents were least affected; those less than 10 years constituted 2.5% while those aged 10-19 years constituted 4.5%. Only 8.5% of the patients were above 70 years of age (Table 1). The commonest symptoms at presentation were fever (94%), cough (92%), shortness of breath (48%) and headache (35%) while the less common symptoms were loss of taste and abdominal pain (0.2%); Table 2. One patient presented with generalized skin rash and one other patient had lymphadenopathy (Table 2).

**Table 1:** sex and age groups of patients (n=200)

variables	Categories	Frequencies (%)
**Sex**	Male	67(33.5)
	Female	133(66.5)
	Total	200(100)
**Age groups**	<10	5(2.5)
	10-19	9(4.5)
	20-29	28(14)
	30-39	40(20)
	40-49	33(16.5)
	50-59	42(21)
	60-69	26(13)
	≥70	17(8.5)
	Total	200(100)

**Table 2:** presenting symptoms of patients and disease severity

variables	Categories	Frequency (%)
**Symptoms**	Fever	94(22.6)
	Cough	92(22.1)
	Shortness of breath	48(11.5)
	Headache	35(8.4)
	Sore throat	31(7.5)
	Anosmia	17(4.1)
	Runny nose	16(3.8)
	Loss of appetite	14(3.4)
	Diarrhochea	12(2.9)
	Weakness	10(2.4)
	Vomiting	7(1.7)
	Malaise	7(1.7)
	Chest pain	6(1.4)
	Myalgia	5(1.2)
	Joint pain	5(1.2)
	Altered consciousness	5(1.2)
	Sputum	4(1.0)
	Fatigue	3(0.7)
	Abdominal pain	1(0.2)
	Skin rash	1(0.2)
	Lymphadenopathy	1(0.2)
	Loss of taste	1(0.2)
	Polydipsia	1(0.2)
**Disease Severity**	Mild	126(63)
	Moderate	22(11)
	Severe	52(26)

**Figure 1:**
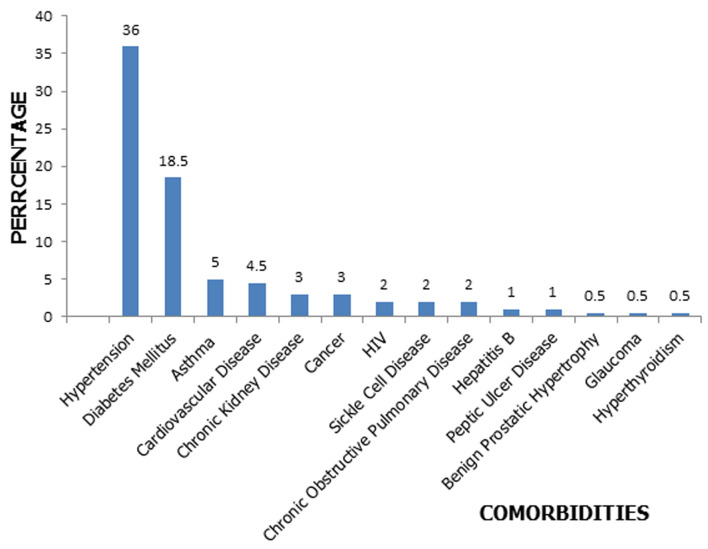
distribution of comorbidities among patients treated for COVID-19

Ninety-four patients (47%) had underlying comorbidities (Figure 1). The commonest was hypertension (36%), followed by diabetes (18.5%), asthma (5%) cardiovascular disease (4.5%), chronic kidney disease (3%), cancer (3%), HIV/AIDS (2%) and sickle cell disease (2%). Less common comorbidities were hepatitis B infection (1%), glaucoma (0.5%) and hyperthyroidism (0.5%) (Figure 1). One hundred and twenty-six patients (63%) had mild disease, 22 (11%) had moderate disease while 52 (26%) had severe disease. All patients with severe disease had oxygen supplementation using nasal prongs or non-rebreather bags. The commonest complication was Acute Respiratory Distress Syndrome (ARDS) seen in 29 (14.5%) patients, followed by Acute Kidney Injury (AKI) seen in 14 (7%) patients. Of those with AKI, 12 (85.7%) were managed conservatively while 2 (14.3%) had renal replacement therapy. Only 2 (1%) patients with ARDS were placed on mechanical ventilator.

Out of the 200 cases managed, 189 (94.5%) were discharged in a stable condition while 11 (5.5%) died. Mortality rates based on disease severity were; 0% (mild disease), 9.1% (moderate disease) and 17.3% (severe disease). Mortality rate for patients with underlying comorbidity was 9.6%, and 1.9% among those that had no underlying comorbidity (Table 3). Patients that had hemodialysis survived while mortality was 100% among those that were placed on mechanical ventilator.

**Table 3:** treatment outcome of patients (n=200) based on age range, presence of comorbidity and disease severity

Variables	Categories	Discharged (n=189)	Dead (n=11)	P-value
Age range	<10	5	0	0.485
	10-19	9	0	
	20-29	28	0	
	30-39	39	1	
	40-49	31	2	
	50-59	39	3	
	60-69	23	3	
	≥70	15	2	
Presence of comorbidity	Yes	94	9	0.017
	No	106	2	
Disease severity	Mild	126	0	0.001
	Moderate	22	2	
	Severe	52	9	

## Discussion

Majority of the patients 133 (66.5%) were males. This is similar to studies conducted in Lagos, Nigeria, which showed that males are more affected than females [10,11]. Preliminary studies in China at the onset of the pandemic showed an even distribution between males and females [14,15]; however, a meta-analysis later in China and studies from the USA showed a male preponderance [16-18]. Studies have shown that women have reduced susceptibility to viral infections [19,20]. This could be attributed to the protection from X chromosome and sex hormones, which play an essential role in innate and adaptive immunity [21]. The mean age of study participants was 45 years similar to a study conducted in the country [11]. The most affected age groups were 30-59 years representing the active workforce in the society contrary to reports from Europe and USA where majority of those affected were elderly people [22,23].

The commonest symptoms at presentation were fever (94%) and cough (92%). Studies from other parts of the world have also shown that fever and cough are the commonest symptoms seen at presentation [7,24,25]. Our findings revealed 16.5% of the patients had digestive symptoms; diarrhea, vomiting, and abdominal pain. Much less than what was reported in a study from China where more than half (50.5%) of the patients diagnosed with COVID-19 had digestive symptoms, mainly diarrhea and anorexia [26]. Infections caused by SARS-CoV-2 may damage the intestinal mucosa and cause digestive symptoms; studies revealed that viral nucleic acid is detected in stool samples in up to 53.4% of patients [27-29]. This shows that though COVID-19 is a respiratory disease, extra pulmonary symptoms are frequently seen. Clinicians must therefore have a high index of suspicion. Only 26% of the patients in this cohort had severe disease. This supports earlier findings that the majority of cases of COVID-19 present as mild disease [10,30].

The presence of underlying comorbidity was seen in 47% of the patients. Previous studies have shown that individuals with underlying comorbidities like cardiovascular disease, hypertension, diabetes, chronic obstructive pulmonary disease (COPD), chronic kidney disease and malignancies are at a greater risk of infection with the SARS-CoV-2 virus [31-33]. The commonest comorbidities were hypertension (36%) and diabetes. Reports have shown increased association between diabetes-related traits and increased ACE2 expression [34]. It has been established that the SARS-CoV-2 virus utilizes ACE-2 receptors, which are found on the surface of the host cells to get inside the cell. ACE-2 breaks down angiotensin-II and to a lesser extent, Angiotensin-I to smaller peptides which play an important anti-inflammatory and anti-oxidant role protecting the lung against ARDS. The use of ACE-2 inhibitors and Angiotensin Receptor Blockers (ARBs) in the treatment of hypertension and diabetes can up regulate the expression of the ACE-2 receptors, thereby leading to increased susceptibility to SARS-CoV-2 infection [35-37]. This may explain why patients with hypertension and diabetes who are on these agents may have an increased susceptibility to COVID-19. Additional factor responsible for increased susceptibility in patients that have diabetes, is the defective phagocytic function of the white blood cells, making them more vulnerable to infections in general [34,38]. In addition, diabetic patients have abundant amount of furin on their cell membranes [39]. This protein activates the spike protein S on the SARS-CoV-2 to bind to the ACE-2 receptors. All these processes and mechanisms make patients with diabetes more vulnerable to COVID-19. Other comorbidities like COPD, chronic kidney disease and malignancy are associated with a weakened immune system and increase susceptibility to infections [31,40,41].

The majority of the patients (94.5%) were discharged home in a stable condition; only 11 (5.5%) out of the 200 patients died. We observed a lower death rate compared to reports from New York with 21% mortality rate [17] and Italy, 26% mortality rate [42]. The lower death rate in this study may be due to the lower age at presentation (median age 45 years) compared to the age at presentation of patients in Europe and USA (63 years) [17,42]. Older patients are associated with more comorbidities and this may also explain the higher death rates among American and European patients with COVID-19.

Even though the overall mortality rate was low (5.5%), the mortality rate among those with severe disease was high (17.3%). There was a significant difference in mortality across the three categories of disease severity, those with severe disease had the highest mortality while those with mild disease had the lowest (p-value=0.001). People with severe disease are more likely to die from COVID-19 than people with mild to moderate disease. Mortality rate was also higher among those with underlying comorbidity (9.6%) compared with those that had no underlying comorbidity (1.9%). This difference was statistically significant (p-value=0.017). The presence of underlying comorbidity was therefore associated with a worse prognosis. This study further buttresses the fact that comorbidities increase the risk of dying from COVID-19.

The limitation of the study is that the retrospective study was not conducted on a large data set over an extended period which could have given a more elaborate view of the descriptive epidemiology of COVID-19. However, this is a novel disease and there was need for an urgent overview of the descriptive epidemiology to broaden the knowledge on the disease in this part of the continent.

## Conclusion

Among Nigerian patients' males are more affected than women while children and adolescents are least affected. The commonest symptoms at presentation are fever, cough, difficulty in breathing, myalgia and headache. Digestive symptoms like diarrhea, vomiting occur commonly. Hypertension, diabetes, cardiovascular diseases, chronic kidney disease and HIV/AIDS are some underlying comorbidities commonly seen. The overall mortality rate is low among Nigerian patients compared to patients in the USA and Europe. Advanced age, presence of underlying comorbidities as well as disease severity is associated with the risk of dying from COVID-19. We recommend a more extensive study of COVID-19 in Nigeria from March 2020 to date (2022) for a more elaborative descriptive epidemiology of the disease.

### What is known about this topic

- Advanced age, presence of underlying comorbidities and disease severity is associated with the risk of dying from COVID-19;
- COVID-19 affects males more than females.

### What this study adds

- The study reveals the clinical features and outcome of coronavirus disease-2019 patients in Abuja, Nigeria;
- The overall mortality rate of coronavirus disease-2019 is low among Nigerian patients compared to patients in the USA and Europe.
